# Ultra-Sensitive, Rapid and On-Site Sensing Harmful Ingredients Used in Aquaculture with Magnetic Fluid SERS

**DOI:** 10.3390/bios12030169

**Published:** 2022-03-09

**Authors:** Meizhen Zhang, Jingru Liao, Xianming Kong, Qian Yu, Miao Zhang, Alan X. Wang

**Affiliations:** 1 School of Petrochemical Engineering, Liaoning Petrochemical University, Fushun 113001, China; zmz121_q@sina.cn (M.Z.); ljru12@163.com (J.L.); qyu@lnpu.edu.cn (Q.Y.); 2 Department of Materials and Environmental Chemistry, Stockholm University, 10691 Stockholm, Sweden; 3 School of Electrical Engineering and Computer Science, Oregon State University, Corvallis, OR 97331, USA; alan.wang@oregonstate.edu

**Keywords:** magnetic fluid, surface-enhanced Raman scattering, POC sensor, AgMNPs, pesticides

## Abstract

The integration of surface-enhanced Raman scattering (SERS) spectroscopy with magnetic fluid provides significant utility in point-of-care (POC) testing applications. Bifunctional magnetic–plasmonic composites have been widely employed as SERS substrates. In this study, a simple and cost-effective approach was developed to synthesize magnetic–plasmonic SERS substrates by decorating silver nanoparticles onto magnetic Fe_3_O_4_ nanoparticles (AgMNPs), which function both as SERS-active substrates and magnetic fluid particles. The strong magnetic responsivity from AgMNPs can isolate, concentrate, and detect target analytes from the irregular surface of fish skin rapidly. We fabricate a microfluid chip with three sample reservoirs that confine AgMNPs into ever smaller volumes under an applied magnetic field, which enhances the SERS signal and improves the detection limit by two orders of magnitude. The magnetic fluid POC sensor successfully detected malachite green from fish with excellent selectivity and high sensitivity down to the picomolar level. This work achieves a label-free, non-destructive optical sensing approach with promising potential for the detection of various harmful ingredients in food or the environment.

## 1. Introduction

Instant and sensitive detection of harmful ingredients in the environment or food is critical for public health to prevent environmental diseases or food-borne illnesses. Various sensing technologies, including gas chromatography−mass spectrometry (GC-MS) [1], electrochemistry [2], photo-electrochemical analysis [3], fluorescence spectroscopy [4], colorimetry [5], and enzyme-linked immunosorbent assay (ELISA) [6] have been developed in past years. Although accurate and reliable in the laboratory, these analytical techniques unfortunately have significant drawbacks, such as long assay times and expensive operational costs. Furthermore, these analytical methods are unsuitable for point-of-care (POC) testing applications due to the complicated sample isolation and bulky instrumentation. It is pivotal to develop a simple, rapid, and cost-effective method for identifying harmful analytes from food and the environment.

Surface-enhanced Raman scattering (SERS) spectroscopy has been recognized as a promising and powerful analytical method that provides the intrinsic molecular information of the analyte with high sensitivity [7,8,9,10]. The advantage of SERS results from the phenomenon that the Raman signal could be significantly enhanced by several orders of magnitude when the molecules are located near or on the surface of the plasmonic substrate [11,12,13,14]. The noninvasive, sensitive, and label-free features of SERS enable them to be widely applied in chemical analysis, environmental protection, biological sensing, biochemical analysis, and food safety [15,16,17,18]. Recent times have also witnessed the innovation of the SERS-based POC sensors as the development of portable Raman spectrometers. Ma’s group developed advanced POC SERS sensors, in which metallic nanoparticles were decorated on the surface of filter paper and used as the sensing chip. The flexible SERS chip successfully detects pesticides from food samples with high sensitivity, and the cost-effective feature of paper shows potential for massive fabrication [19,20]. Tran et al. designed and fabricated a portable SERS device for a rapid lateral flow assay (LFA). The POC sensor can detect the pregnancy hormone human chorionic gonadotropin in 5 s, and the sensitivity is almost 15 times higher than the commercial LFA [21]. The conventional SERS substrates in POC devices are Ag or Au nanomaterials, in which the metallic nanostructure could provide a prominent enhancement effect. Despite the significant SERS sensitivity, colloidal Ag or Au nanostructures still have challenges in POC SERS detection. First, the complex components in target samples easily interfere with SERS measurement. Therefore, the isolation step of analytes is indispensable before SERS measurement. Second, the precise control of analytes within a special area during Raman measurement can overcome the uncontrollable distribution of plasmonic nanomaterials.

Development in the fields of microfluidics has accelerated the progress of magnetic fluid devices. The magnetic–plasmonic composite and microfluidic chip are integrated to synergistically provide advanced properties in POC SERS sensors, which are well-suited for chemical detection and food safety monitoring [22,23,24,25]. The Fe_3_O_4_ NPs are commonly used as a magnetic core to construct plasmonic–magnetic composites in SERS sensing due to their excellent magnetic responsibility and stability [26]. By combining the SERS activity of noble metallic NPs and the sample separation capability of magnetic Fe_3_O_4_ NPs, these magnetic–plasmonic SERS substrates could be employed to isolate analytes from complex samples by applying magnetic fields and working as active substrates to enhance the SERS signals of the target analyte. In addition, the microfluidic chip offers attractive advantages, including in-built micromixers and rapid detection, which are suitable for on-site monitoring of harmful ingredients from environmental or food samples. 

**Scheme 1:**
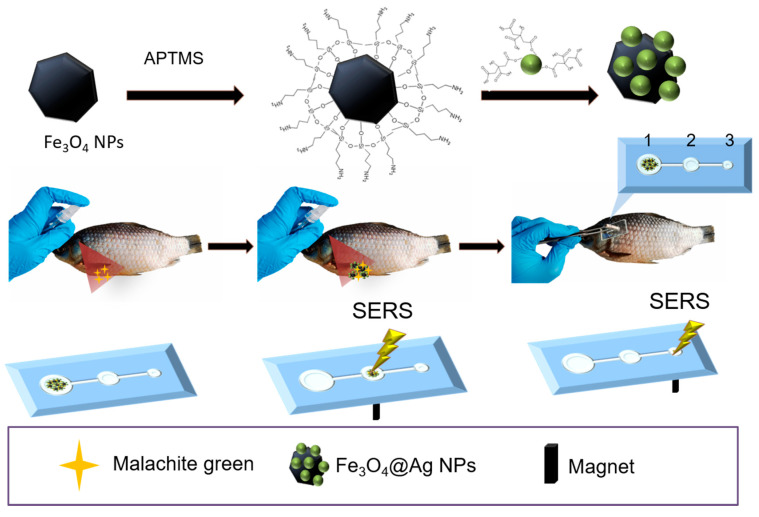
Schematic illustration of the synthesized magnetic–plasmonic composite and its application for sensing MG from fish skin by magnetic fluid SERS.

In this study, we designed and fabricated a fast-responsive smart method for controllable mass transport and distribution by integrating magnetic fluid into the microchip. The AgMNPs were fabricated by decorating Fe_3_O_4_ with Ag NPs, in which the Fe_3_O_4_ NPs were commercially available. The magnetic AgMNPs presented high magnetic responsibility and excellent SERS activity. The microfluid chip confines the magnetic–plasmonic composite into cascaded reservoirs with ever smaller volumes under an external magnetic field, which could concentrate the target analyte and further enhance the SERS signal, as shown in Scheme 1. The proposed magnetic-fluidic device successfully detected malachite green (MG) from the fish skin, and the sensitivity reached the picomolar level. The results indicate that the microfluid chip with AgMNPs as magnetic fluid carriers possesses good sensitivity and selectivity in food safety and shows great potential for practical POC application in real applications.

## 2. Experimental Section

### 2.1. Chemicals and Reagents

Magnetic Fe_3_O_4_, MG, and 3-aminopropyltrimethoxysilane (APTMS) were obtained from Aladdin (Shanghai, China). AgNO_3_ and p-aminothiophenol (PATP) were supplied by Innochem (Beijing, China). Sodium citrate was purchased from Macklin (Shanghai, China). The fish used in the experiment were purchased from local supermarkets. The water used in this research was double-distilled water. All reagents used in the experiment were analytical grade and used without further purification.

### 2.2. Synthesis of Ag Colloids

AgNO_3_ (0.034 g) was dissolved in 200 mL of ultrapure water and heated to reflux. Then, 4 mL of sodium citrate solution (1% *w*/*v*) was used as a reductant and quickly added into the boiling solution with vigorous stirring. The mixture was further heated at reflux for 1 h. Finally, the Ag colloid with a yellow–green appearance was cooled to room temperature and kept at 4 °C for further use. 

### 2.3. Fabrication of Magnetic–Plasmonic AgMNPs

The magnetic–plasmonic composites (AgMNPs) were fabricated through a traditional self-assembly method. Five milligrams of Fe_3_O_4_ was dispersed in 10 mL of ethanol, and then 5 μL of APTMS were dropped into the suspension. The mixture was ultrasonicated for 5 min. After that, the mixture was kept rotation for 6 h under ambient conditions, and then heated at 70 °C for 10 min. The APTMS modified Fe_3_O_4_ NPs were washed thoroughly with ethanol and water five times, and then dispersed with 4 mL of Ag colloid. The mixture was ultrasonicated for 10 min and rotated for another 2 h, the magnetic–plasmonic AgMNPs were separated by an applied magnetic field and dispersed in 100 µL water.

### 2.4. Chip Design and Fabrication

The magnetic fluid chip was designed to maneuver the magnetic–plasmonic composite through the process of separation, concentration, and SERS detection. The microfluid chip has a channel length of 3 cm, with three cascaded reservoirs. The reservoirs have diameters at 5 mm, 2 mm, and 1 mm, respectively. The glass microchip was fabricated using Microflutech. Co., Ltd. (Changzhou, China).

### 2.5. SERS Measurement

The SERS activity of AgMNPs was characterized with PATP, a typical Raman probe molecule. The AgMNPs were dispersed in 10^−5^ M of ethanol solution of PATP. After shaking for 1 h, the AgMNPs were adsorbed by the magnetic field and transferred onto the microfluid chip by placing a strong magnet at the back of the glass slide. Raman spectra were acquired by a portable Raman spectrometer (BWS465-785S; B&W Tek, Newark, DE, USA) equipped with a 785 nm laser as the excitation light. The resolution of the spectra was set as 5 cm^−1^ and the diameter of the laser spot was 105 μm. All Raman spectra were measured in the range from 300–3000 cm^−1^. The data were collected using BWSPEC software. For on-site detection of pesticides from fish, 1 mL of aqueous solution of MG at different concentrations was sprayed onto the fish skin (4 × 4 cm). Then, the AgMNPs suspension was directly sprayed onto the surface of the fish and transferred to the microfluid chip under a magnetic field, followed by Raman measurement.

### 2.6. Other Measurements

Scanning electron microscopic (SEM) photographs and energy dispersive spectroscopy (EDS) were collected on a SU 8010 field emission scanning electron microscope (Hitachi, Japan). The UV–vis absorption spectra of AgMNPs were measured on a UV 2400 UV–vis spectrophotometer (Sunny Hengping Instrument, Shanghai, China). The X-ray diffraction (XRD) analysis of the Fe_3_O_4_ and AgMNPs was developed on a D8 Advance X-ray powder diffractometer (Bruker, Germany). The magnetic loop of the magnetic–plasmonic composite was obtained from an MPMS SQUID VSM (Quantum Design, San Diego, CA, USA). The fluorescence microscopic images were collected using a fully automated Nikon inverted microscope Eclipse Ti2-E (Nikon, Tokyo, Japan). The wavelength of the excitation light was 561 nm.

## 3. Results and Discussion

### 3.1. Construction and Characterization of AgMNPs

**Figure 1:**
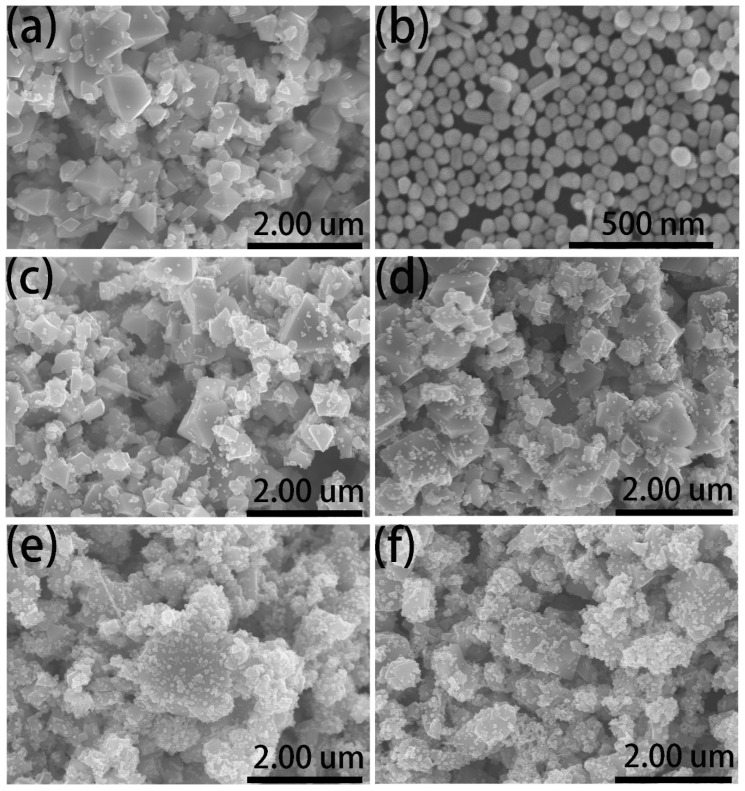
SEM images of (**a**) Fe_3_O_4_, (**b**) Ag NPs, and AgMNPs prepared with Ag colloid concentrated at different times (**c**) original Ag colloid, (**d**) 4 times, (**e**) 8 times, and (**f**)16 times.

The process to synthesize the magnetic–plasmonic composite is presented in Scheme 1. The Fe_3_O_4_ NPs were used as the magnetic core in the magnetic–plasmonic composite and were modified with APTMS. The amino group on the surface of Fe_3_O_4_ APTMS provides positive charges. The plasmonic Ag NPs were synthesized through the citrate sodium method, and the surface of Ag NP showed negative charges as the carboxylate group of the citrate. The magnetic–plasmonic AgMNPs were constructed via the assembly of Ag NPs onto the surfaces of Fe_3_O_4_ by electrostatic interaction. The MNPs used in this research were commercially available Fe_3_O_4_ powder, which is very cost-effective compared with laboratory-prepared materials. The SEM image of Fe_3_O_4_ MNPs is shown in Figure 1a. The diameters were between 200 and 1000 nm. The feature of plasmonic NPs used for synthesizing the magnetic–plasmonic AgMNP substrate is a crucial factor for the performance in SERS detection. The morphology and diameter of Ag NPs were determined by the SEM image, as shown in Figure 1b. The size of Ag NPs was distributed between 50 and 70 nm, and the mean size was nearly 60 nm, which is suitable for SERS enhancement. After mixing Fe_3_O_4_-APTMS with Ag colloids, magnetic–plasmonic AgMNPs were formed. Figure 1c–f present the SEM images of AgMNPs assembled in different concentrations of Ag colloids, in which the Ag NPs were distributed on the surface of Fe_3_O_4_. Furthermore, with the increase in Ag colloid concentration, the density of Ag NPs on Fe_3_O_4_ was consequently increased. The density of Ag NPs is low when the original Ag colloid is used for self-assembly, as shown in Figure 1c. In order to obtain dense Ag NPs on the Fe_3_O_4_, the Ag colloids were concentrated 4, 8, and 16 times through centrifuge. The Ag NP density on the surface of Fe_3_O_4_ greatly increased as the Ag colloid concentrated 8 and 16 times.

In order to determine the surface chemical composition, AgMNPs were examined by EDS spectra, as presented in Figure S1, in which the elemental Fe, O, and Ag were detected. The ratio of Ag was 2.32%, as the original Ag colloid used for self-assembly. When the Ag colloid was concentrated 8 times, the ratio of Ag in the AgMNPs increased to 14.48%. EDS elemental mapping images of AgMNPs are shown in Figure S2. It can be observed from the elemental mapping image that Fe and Ag elements are clearly presented on the magnetic–plasmonic materials; meanwhile, Fe and Ag elements are uniformly distributed on AgMNPs. The elemental Ag in the image was increased as the concentration of Ag colloid increased from 1 to 8 times. These results indicated that the density of Ag on the magnetic–plasmonic material could be controlled easily by adjusting the concentration of Ag colloid.

**Figure 2:**
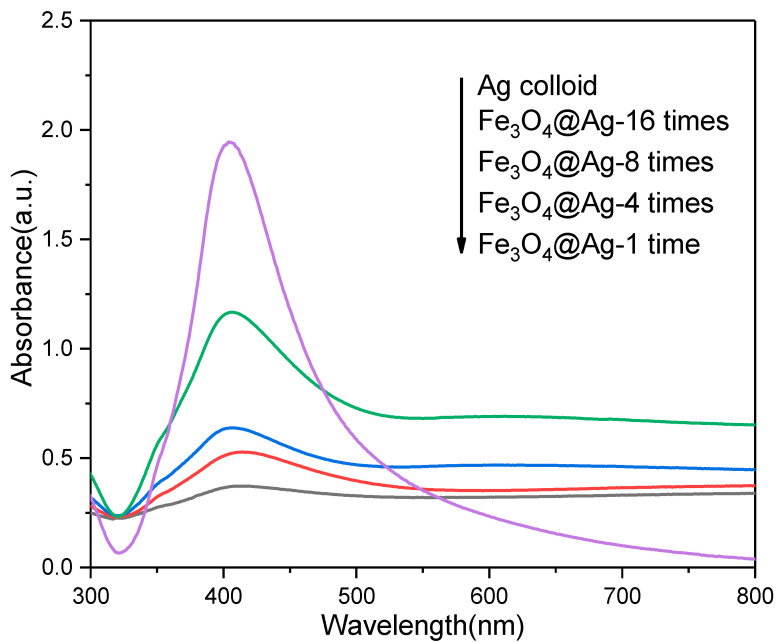
UV-vis absorption spectra of Ag colloid and AgMNPs prepared with different concentrations of Ag colloid.

The UV-vis spectra were employed for measuring plasmonic features of the Ag colloid and AgMNPs, as shown in Figure 2. The maximum absorption band of the Ag colloid was located at 403 nm due to the localized surface plasmon resonance (LSPR) of Ag NPs [27]. The LSPR band of metallic NPs is highly related to the shape, diameter, and environment around the NPs [28]. The UV-vis absorption spectra indicate that the Ag NPs were monodispersed and the diameter ranged from 40 to 60 nm. The UV–vis absorption spectra of AgMNPs assembled with different concentrations of Ag colloid are shown in Figure 2. The obvious adsorption bands located at around 405 nm were assigned to the LSPR of the spherical Ag NPs in AgMNPs. Furthermore, as the concentration of Ag colloids used in the self-assemble process increases, the intensity of absorption peaks of AgMNPs gradually increases as well. The results coincide with the SEM images, which further demonstrate that the amount of Ag NPs on Fe_3_O_4_ was proportional to the concentration of Ag colloid.

**Figure 3:**
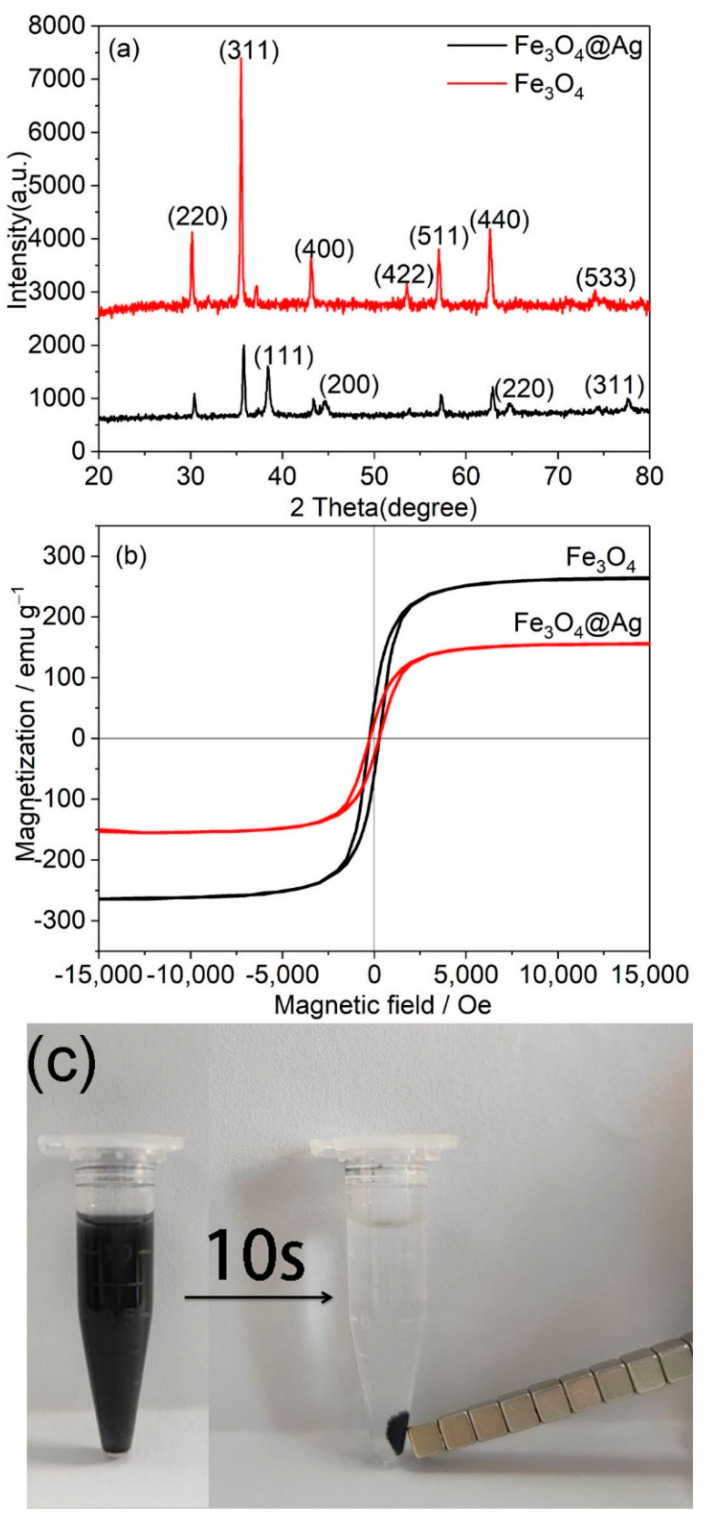
(**a**) XRD patterns of Fe_3_O_4_ and AgMNPs. (**b**) Magnetic hysteresis curves of Fe_3_O_4_ and AgMNPs and (**c**) the separation property of AgMNPs under an applied magnetic field.

The composition and phase purity of the magnetic–plasmonic composite were characterized by XRD, as shown in Figure 3a. The Fe_3_O_4_ presented diffraction peaks at 30.2°, 35.5°, 43°, 53.5°, 56.9°, 62.5°, and 74°, which are marked with their indices 220, 311, 400, 422, 511, 440, and 533 (JCPDS Card No. 19-0629) [29]. The XRD results indicated that the magnetic powder was pure Fe_3_O_4_ with an inverse cubic spinal structure. After decorating Ag NPs on the surface of Fe_3_O_4_, four new diffraction peaks at 2θ = 38.1°, 44°, 65°, and 77.5° were observed, which were assigned to 111, 200, 220, and 311 planes of Ag crystalline structure (JCPDS card No. 87-0597) [30]. The results indicated that the crystalline Ag NPs were successfully decorated on the surface of Fe_3_O_4_. The magnetic features of the magnetic–plasmonic composites are shown in Figure 3b. The saturation magnetization (MS) values of the Fe_3_O_4_ and AgMNPs were 266 emu/g and 157 emu/g, respectively. They exhibited little hysteresis and coercivity of magnetic particles. The MS value of the AgMNPs decreased after the decoration of the Ag NPs. However, the MS value of AgMNPs was maintained at a high level, which enabled the AgMNPs to instantly isolate from the target system within only 10 s with the application of an external magnetic field, as shown in Figure 3c. These results indicated that the as-prepared AgMNPs could be used as an efficient magnetic–plasmonic composite for the rapid separation of analyte from the sample solution.

### 3.2. SERS Performance of the AgMNPs

**Figure 4:**
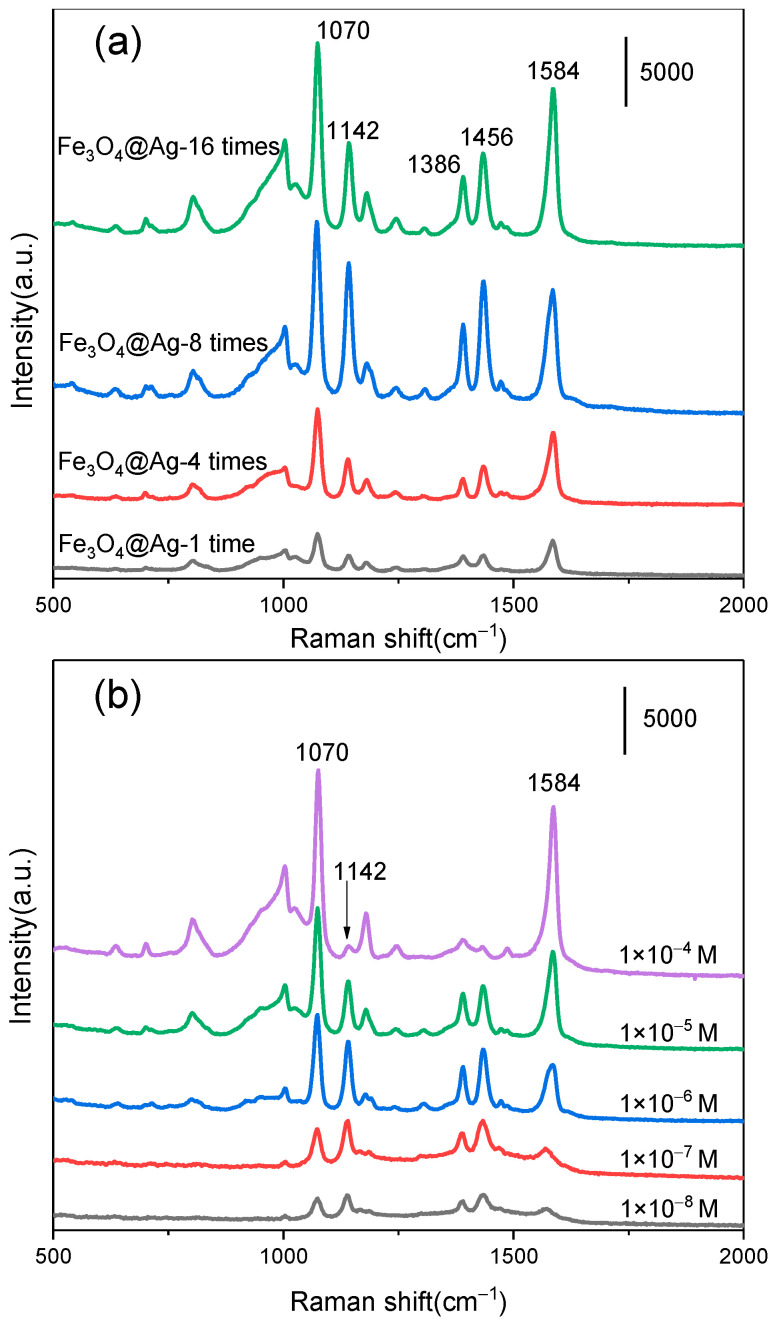
Raman spectra of PATP on the prepared AgMNPs with different concentrations of Ag colloid (**a**), and SERS spectra of different concentrations of PATP adsorbed on AgMNPs (**b**).

The density of Ag NPs decorated on Fe_3_O_4_ was highly related to the SERS activity of the magnetic–plasmonic composite. The SERS activity of the AgMNPs was optimized by using PATP as a Raman probe molecule. The AgMNPs were first immersed in an aqueous solution of PATP (10^−4^ M) for 2 min and separated by the magnetic field. The SERS spectra of PATP measured from AgMNPs assembled in the Ag colloid with different concentrations are shown in Figure 4. SERS peaks at 1070 cm^−1^, 1142 cm^−1^, 1386 cm^−1^, 1436 cm^−1^, and 1584 cm^−1^ were observed. The Raman spectra of solid PATP are presented in Figure S3, in which only two prominent peaks were observed. The peaks at 1070 cm^−1^ and 1584 cm^−1^ were assigned to the a1 vibration of PATP (in-plane) [31]. The SERS peaks of the aqueous solution of PATP differed from the Raman spectra of solid PATP, which were due to the new molecule (4,4′-dimercaptoazobenzene, DMAB) formed. The photocatalytic reaction occurred during the Raman measurement process, in which the PATP was coupled to each other on the plasmonic Ag NPs under laser irradiation [32]. The new Raman peak at 1142 cm^−1^ is attributed to the vibration of β(CH) of the DMAB, and the new bands at 1386 cm^−1^ and 1436 cm^−1^ are assigned to the stretching vibration of the N-N group in DMAB. The AgMNPs assembled in the original Ag colloid provided a weak SERS signal. As shown in Figure 4, the intensity of the SERS signals increased as the concentrations of Ag colloids increased. The AgMNPs provided intense SERS signal of DMAB when the Ag colloid was concentrated by 8 times. Further concentrating the Ag colloid to 16 times, however, shows a weaker SERS signal from AgMNPs compared with the 8 times concentration. The 8 times concentrated Ag colloids were chosen in the assembled process for constructing magnetic–plasmonic AgMNPs. The SERS performance of the AgMNPs was also evaluated by using PATP as a probe molecule. Figure 4b presents the SERS signal of PATP at concentrations from 10^−4^ M to 10^−8^ M. The intensities of the SERS spectra were weakened with the decrease of PATP concentration. However, the obvious SERS spectra were still obtained as the concentration down to 10^−8^ M. The SERS spectra gradually changed as the concentration of PATP decreased. A weak peak at 1142 cm^−1^ assigned to DMAB was observed when the concentration of PATP was 10^−4^ M. This result indicated that the dominant species on the surface of the AgMNPs was PATP. The reason was that the multilayer PATP was decorated on the surface of the plasmonic SERS substrate as a high concentration of PATP was used; only a small part of PATP was coupled to DMAB as the laser illuminated. As the concentration of PATP decreased, the intensity ratio between the peak at 1142 cm^−1^ and 1086 cm^−1^ was increased obviously, which indicated that the proportion of DMAB on the surface of AgMNPs increased.

### 3.3. Sample Enrichment Effect of the Magnetic Fluid Device

**Figure 5:**
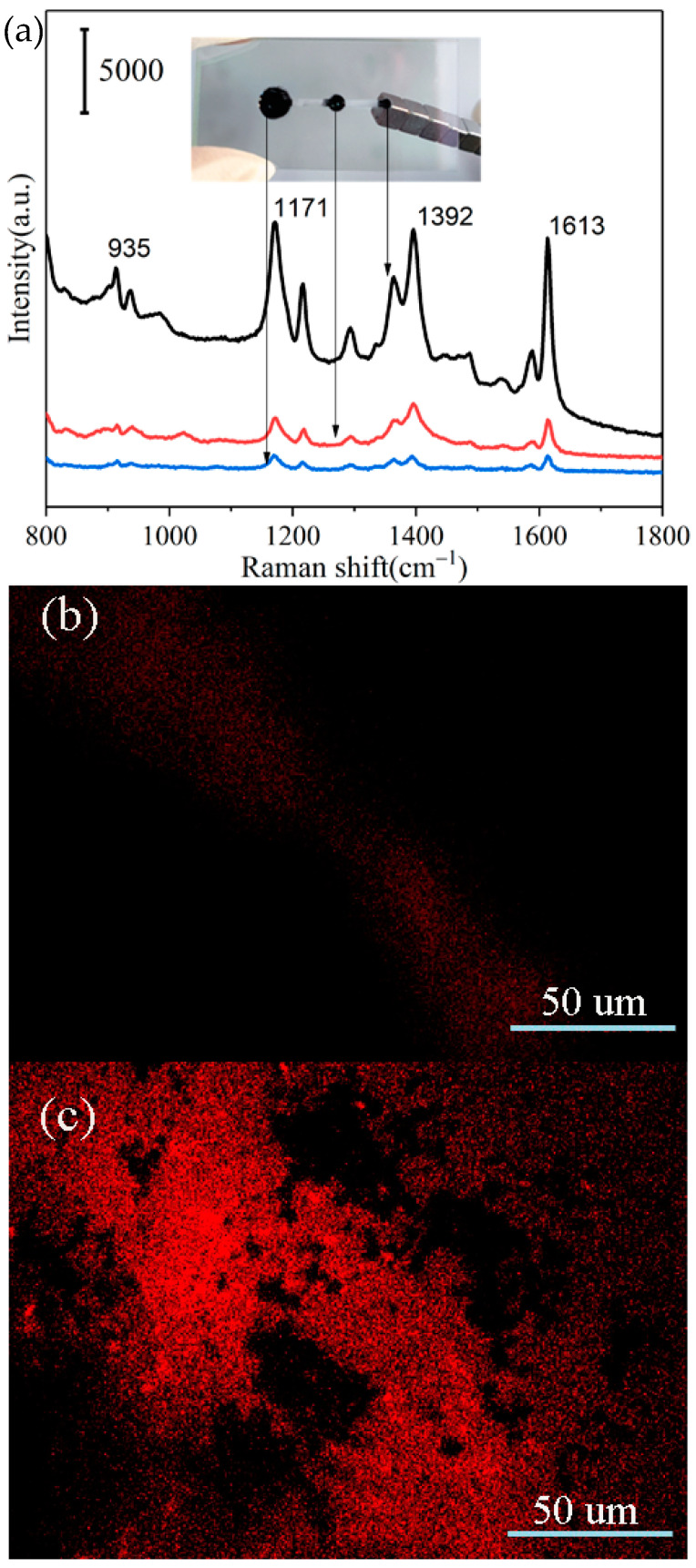
Photographic image of the microfluidic channel and corresponding SERS spectra at three reservoirs (**a**); fluorescence microscopic image of Rhodamine 6G (5 ppm) on AgMNPs from reservoir 1 (**b**) and reservoir 3 (**c**).

**Figure 6:**
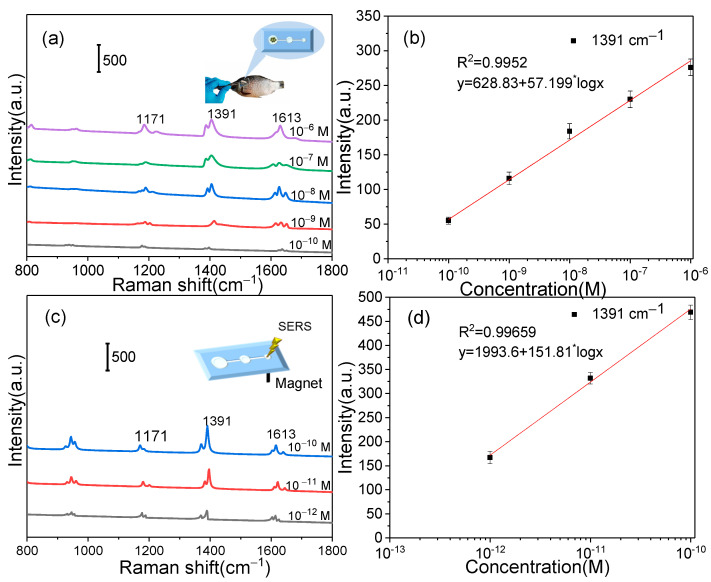
SERS spectra of MG measured from reservoir 1 (**a**) and reservoir 3 (**c**) by magnetic-fluidic SERS method, the corresponding dose-response curves of the Raman peaks at 1391 cm^−1^ (**b**,**d**).

The microchip plays an important role in the sample enrichment for SERS detection by using the magnetic–plasmonic composite, in which 3 sample reservoirs with different diameters were distributed on the chip, as shown in Figure 5a. The sample enrichment effect of the microchip integrated with the magnetic–plasmonic composite was characterized by the SERS spectra of MG (10^−6^ M). Ten microliters of AgMNPs (1 mg/mL) was dropped onto the reservoirs of the microchip. The SERS signal of MG was obtained from reservoir 1, as shown in Figure 6. The characteristic Raman peaks of the MG were observed at 935 cm^−1^, 1171 cm^−1^, 1392 cm^−1^, and 1613 cm^−1^. The peak at 935 cm^−1^ is due to the ring skeletal vibration of radical orientation, the intense peak at 1171 cm^−1^ is attributed to the in-plane vibration of the aromatic ring, the peak at 1392 cm^−1^ is assigned to the symmetric stretching vibration of the C–N group, and the intense peak at 1613 cm^−1^ is attributed to the stretching vibration of the aromatic ring [33]. The AgMNPs adsorbed with MG were loosely distributed on the surface of reservoir 1 due to the large surface area. The SERS spectra of MG from reservoir 2 are more intense than those from reservoir 1 and the reason was the intensity of Raman spectra depends on the number of analytes. The smaller diameter of reservoir 2 (2 mm) could confine the AgMNPs SERS substrate to a smaller area, which increased the density of analytes within the certain area. In order to further increase the intensity of the SERS signal from the microchip, the magnetic tip was applied under the third reservoir to concentrate the AgMNPs. The most intense SERS signal was obtained from reservoir 3, which was enhanced almost 10 times when compared with reservoir 2. The significant enhancement could be assigned to two aspects. First, reservoir 3 could confine the AgMNPs within a small area. The surface area of reservoir 3 is only one-quarter of reservoir 2. Second, the applied magnetic tip could make the AgMNPs aggregate together; the analytes on the SERS substrate are concentrated to a smaller area, especially when more SERS hotspots were formed during the aggregation of AgMNPs, which effectively increased the SERS signal and improved the sensitivity in detection.

The concentration effect of the method was investigated using fluorescence microscopy. First, the AgMNPs (2 mg) were added to 1 mL aqueous solution of Rhodamine 6G(R6G) (5 ppm) for 2 min and then separated with magnetic field and washed with water. The AgMNPs with R6G were dispersed with 1 mL of water, and 5 µL suspension of AgMNPs were dropped into reservoir 1 and reservoir 3, respectively. The fluorescence microscopic images were collected from reservoir 1 and reservoir 3 and are shown in Figure 5a,b. The fluorescence microscopic image exhibited an obvious contrast between reservoir 1 and reservoir 3. An intensely bright fluorescence image was observed from reservoir 3. These results confirm that a high density of AgMNPs was confined in reservoir 3, which led to a higher analyte concentration in the sample reservoir with a small area.

### 3.4. Magnetic-Fluidic SERS Sensing MG from the Fish Sample

MG, a triphenylmethane dye, has been employed as a fungicide in aquaculture due to its high antibacterial effect. Due to its genotoxic and carcinogenic properties, MG is forbidden for aquaculture in China, the European Union, the United States, and many other countries. A magnetic fluid device was employed for sensing MG from fish samples by SERS. The AgMNPs were first sprayed on fish skin contaminated with different concentrations of MG. Then, the microchip was swabbed from the surface of fish with an applied magnetic field under reservoir 1, and the AgMNPs were adsorbed on the microchip during the swabbing process. The SERS spectra of MG from reservoir 1 are shown in Figure 6a, the characteristic peaks of MG were observed. The intensity of the Raman signal of MG decreased as the decrease of MG concentrations from 10 × 10^−6^ M to 10 × 10^−10^ M (Figure 6a). There were nearly no feature Raman peaks of MG observed from reservoir 1 when the concentration was down to 10^−10^ M. As the magnetic field was applied to transfer the AgMNPs to reservoir 3 on the fluidic chip, the SERS spectra were measured and presented in Figure 6c. The feature Raman peaks of MG were measured even though the concentration was down to 10^−12^ M, which indicated the good SERS sensitivity of the magnetic fluid device. The corresponding linear relationship between the intensity of Raman signals (1391 cm^−1^) and the concentration of MG is shown in Figure 6b,d. The coefficient of linear relationship (R^2^) was 0.9952 and 0.9966, corresponding to reservoir 1 and reservoir 3, respectively. 

**Figure 7:**
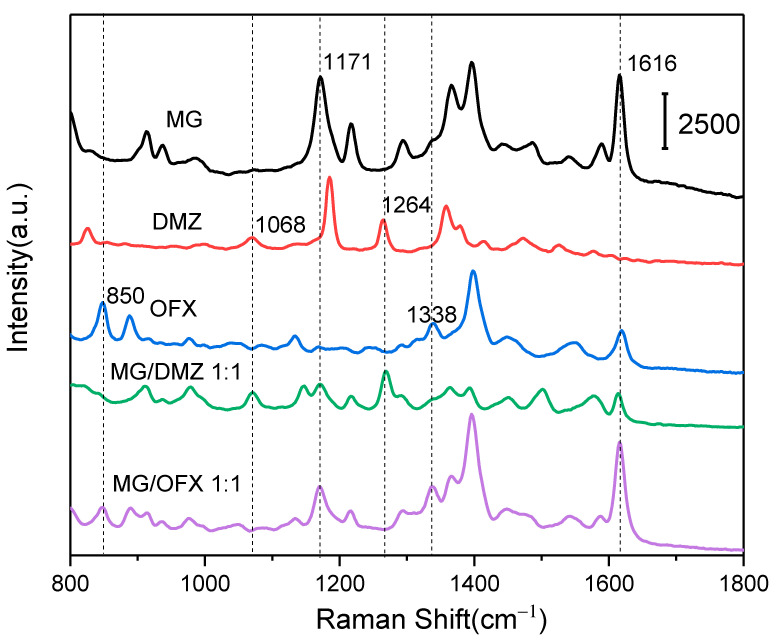
Multiplex detection of MG from mixtures.

In real applications, mixed pesticides are usually applied in aquaculture or agriculture. Multiple pesticides would be present in the fish simultaneously. In order to investigate the selectivity of the magnetic fluid device, dimetridazole and ofloxacin were mixed with MG to construct mixture samples. The SERS spectra corresponding to MG, dimetridazole, ofloxacin, and their mixtures are shown in Figure 7, in which the feature Raman peaks of the pure substance are labeled. The characteristic Raman peak of the pure substances was measured from two mixtures, which indicated that the AgMNPs SERS composite has excellent selectivity to MG in SERS sensing.

## 4. Conclusions

In summary, we have proposed a new type of magnetic-fluidic SERS sensing device that was successfully applied for instant, ultra-sensitive, label-free, and on-site identification of pesticides from fish. The magnetic–plasmonic composites (AgMNPs) were synthesized by controllable assembling Ag NPs onto Fe_3_O_4_, which was cost-effective as the magnetic particles are commercial Fe_3_O_4_ powder at low prices. The AgMNPs function as a multifunctional composite for the isolation and sample enrichment of target analytes from fish, and SERS identification of such pesticides on the fluidic microchip. Furthermore, the fluidic microchip showed highly effective confinement of the AgMNPs as the ultra-small dimension of the sample reservoir on the chip. The magnetic–plasmonic fluid was confined in different sample reservoirs under an applied magnetic field. Based on this strategy, the detection sensitivity of MG from fish was achieved to picomolar, which was nearly two orders of magnitude better compared with normal SERS methods [34]. The device also exhibited excellent capability for detecting multiplex pesticides from mixtures. Such a facile magnetic fluid device using plasmonic AgMNPs, as a new kind of cheap and ultra-sensitive on-chip device with multiplex detecting capabilities, will have significant potential application in chemical analysis and food safety.

## Data Availability

The data presented in this study are available on request from the corresponding author.

## Supplementary Materials

The following are available online at https://www.mdpi.com/article/10.3390/bios12030169/s1, Figure S1: EDS spectra of AgMNPs prepared with original Ag colloid (a) 8 times concentrated Ag colloid (b), Figure S2: Elemental mappings of AgMNPs prepared with original Ag colloid (a,b) 8 times concentrated Ag colloid (c,d). Figure S3: Raman spectra of solid PATP.
