# Supplementary material for: Ultra-Sensitive, Rapid and On-Site Sensing Harmful Ingredients Used in Aquaculture with Magnetic Fluid SERS
Source: Biosensors (Basel). 2022 Mar 9;12(3):169. doi: 10.3390/bios12030169 (PMC8946156; doi:10.3390/bios12030169)
Supplement: Supplementary file 1 [file biosensors-12-00169-s001.zip › biosensors-1618613-supplementary.pdf]

biosensors

MDPI

Article

Ultra-Sensitive, Rapid and On-Site Sensing Harmful Ingredients Used in Aquaculture with Magnetic Fluid SERS

Meizhen Zhang [1], Jingru Liao [1], Xianming Kong [1,*], Qian Yu [1], Miao Zhang [2,*] and Alan X. Wang [3]

[1] School of Petrochemical Engineering, Liaoning Petrochemical University, Fushun 113001, China; zmz121_q@sina.cn (M.Z.); ljru12@163.com (J.L.); qyu@lnpu.edu.cn (Q.Y.)
[2] Department of Materials and Environmental Chemistry, Stockholm University, 10691 Stockholm, Sweden
[3] School of Electrical Engineering and Computer Science, Oregon State University, Corvallis, OR 97331, USA; alan.wang@oregonstate.edu
* Correspondence: xmkong@lnpu.edu.cn (X.K.); miao.zhang@mmk.su.se (M.Z.)

Citation: Zhang, M.; Liao, J.; Kong, X.; Yu, Q.; Zhang, M.; Wang, A.X. Ultra-Sensitive, Rapid and On-Site Sensing Harmful Ingredients Used in Aquaculture with Magnetic Fluid SERS. *Biosensors* **2022**, *12*, 169. https://doi.org/10.3390/bios12030169

Received: 15 February 2022
Accepted: 7 March 2022
Published: 9 March 2022

Publisher's Note: MDPI stays neutral with regard to jurisdictional claims in published maps and institutional affiliations.

Copyright: © 2022 by the authors. Licensee MDPI, Basel, Switzerland. This article is an open access article distributed under the terms and conditions of the Creative Commons Attribution (CC BY) license (https://creativecommons.org/licenses/by/4.0/).

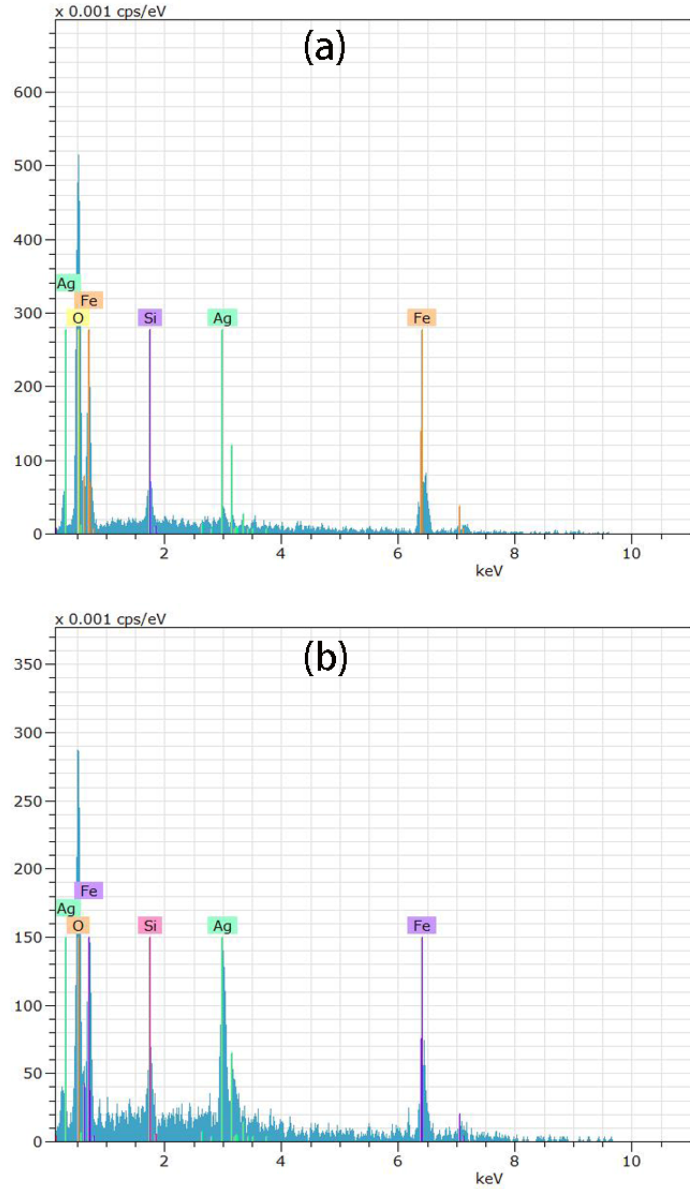

Figure S1. EDS spectra of AgMNPs prepared with original Ag colloid (a) 8 times concentrated Ag colloid (b).

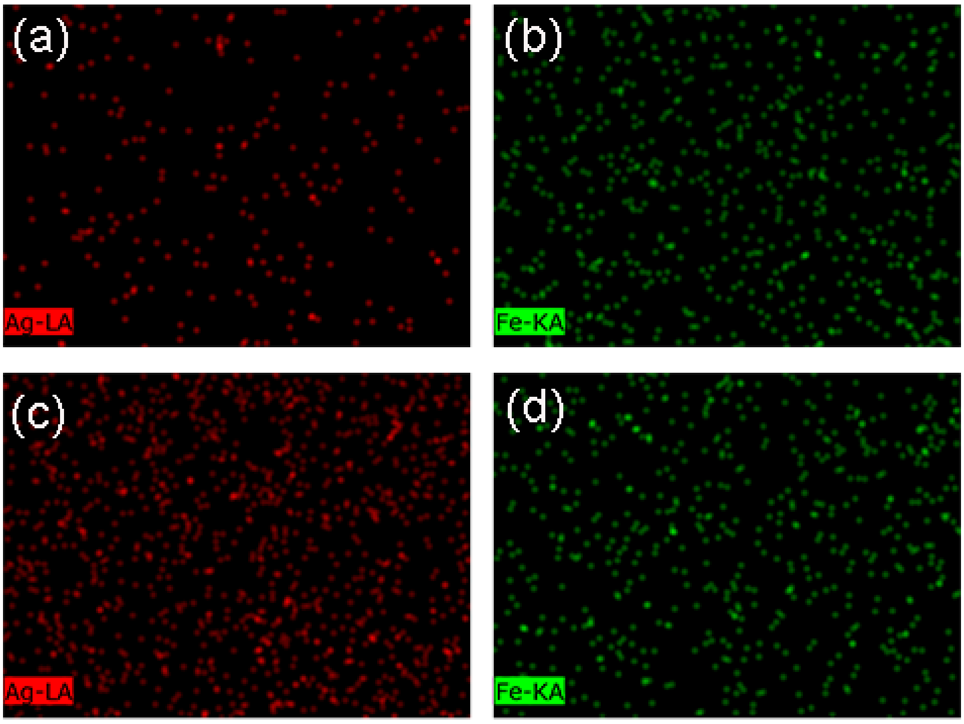

Figure S2. Elemental mappings of AgMNPs prepared with original Ag colloid (a, b) 8 times concentrated Ag colloid (c, d).

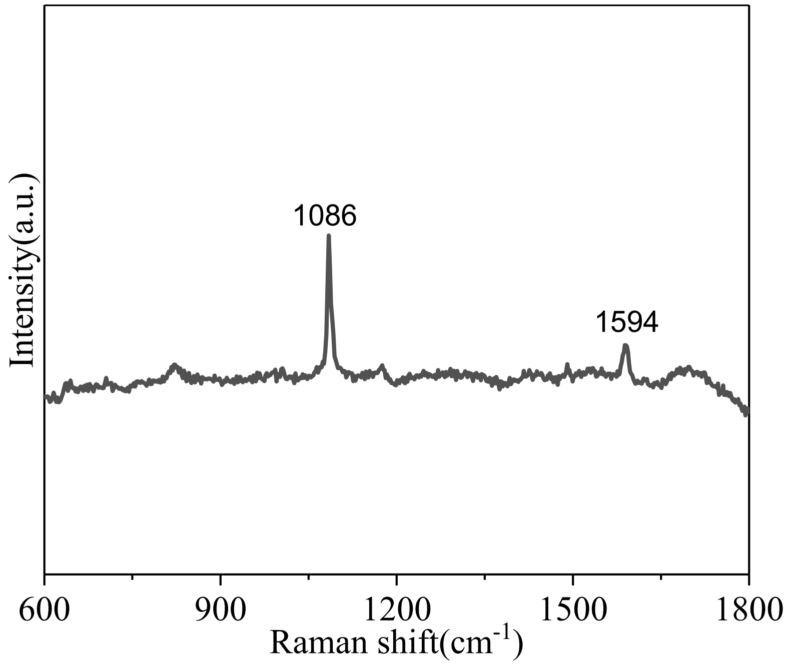

Figure S3. Raman spectra of solid PATP.
